# Early Post Operative Enteral Versus Parenteral Feeding after Esophageal Cancer Surgery 

**Published:** 2015-09

**Authors:** Mohammad Taghi Rajabi Mashhadi, Reza Bagheri, Majid Ghayour-Mobarhan, Marzie Zilaee, Reza Rezaei, Ghodratollah Maddah, Mohamad Reza Majidi, Mojgan Bahadornia

**Affiliations:** 1 *Endoscopic & Minimally Invasive Surgery Research Center, Mashhad University of Medical Sciences, Mashhad, Iran.*; 2 *Cardio-Thoracic Surgery & Transplant Research Center, Emam Reza Hospital, Faculty of Medicine, Mashhad University of Medical Sciences, Mashhad, Iran.*; 3 *Biochemistry of Nutrition Research Center, School of Medicine, Mashhad University of Medical Sciences, Mashhad, Iran.*; 4 *Department of Nutrition, Faculty of Paramedicine, Ahvaz Jundishapur University of Medical Sciences, Ahvaz, Iran.*; 5 *Department of General Surgery, Ghaem Hospital, School of Medicine, Mashhad University of Medical Sciences, Mashhad, Iran.*; 6 *Sinus and Surgical Endoscopic Research Center, Ghaem Hospital, Faculty of Medicine, Mashhad University of Medical Sciences, Mashhad, Iran*

**Keywords:** Esophageal cancer, Enteral feeding, Parenteral feeding, Nutritional assessment

## Abstract

**Introduction:**

The incidence of malnutrition in hospitalized patients is reported to be high. In particular, patients with esophageal cancer are prone to malnutrition, due to preoperative digestive system dysfunctions and short-term non-oral feeding postoperatively. Selection of an appropriate method for feeding in the postoperative period is important in these patients.

**Materials and Methods:**

In this randomized clinical trial, 40 patients with esophageal cancer who had undergone esophagectomy between September 2008 and October 2009 were randomly assigned into either enteral feeding or parenteral feeding groups, with the same calorie intake in each group. The level of serum total protein, albumin, prealbumin, transferrin, C3, C4 and hs-C-reactive protein (hs-CRP), as well as the rate of surgical complications, restoration of bowel movements and cost was assessed in each group.

**Results:**

Our results showed that there was no significant difference between the groups in terms of serum albumin, prealbumin or transferrin. However, C3 and C4 levels were significantly higher in the enteral feeding group compared with the parenteral group, while hs-CRP level was significantly lower in the enteral feeding group. Bowel movements were restored sooner and costs of treatment were lower in the enteral group. Postoperative complications did not differ significantly between the groups. There was one death in the parenteral group 10 days after surgery due to myocardial infarction.

**Conclusion:**

The results of our study showed that enteral feeding can be used effectively in the first days after surgery, with few early complications and similar nutritional outcomes compared with the parenteral method. Enteral feeding was associated with reduced inflammation and was associated with an improvement in immunological responses, quicker return of bowel movements, and reduced costs in comparison with parenteral feeding.

## Introduction

Malnutrition is defined as a state in which a deficiency of nutrients such as energy, protein, vitamins and minerals causes measurable adverse effects on body composition, function or clinical outcome (National Institute of Clinical Excellence (NICE) guideline (2006).

The incidence of malnutrition in hospitalized patients has been reported to be over 50%, and exists even in the best treatment centers. Patients with esophageal cancer often have malnutrition caused by dysphagia and malignant cachexia, and short-term (7–10 days), non-oral feeding after surgery accentuates this problem (1). Identifying an appropriate nutritional method that is cost effective, physiological and that preserves the immune response in the postoperative period is clearly important. There are currently controversies concerning the optimal method of feeding in the postoperative period among patients with esophageal cancer (2,3). Cancer cachexia is a multi-factorial condition and results in weight loss and muscle atrophy, disturbance of the immunologic system and metabolic changes. Patients with cancer and malnutrition do not respond well to common methods of treatment(surgery-chemotherapy or radiation therapy) (4). Malnutrition also increases the rate of complications including infection, hospitalization, and mortality rate as well as treatment costs. In fact, a serum albumin level less than 29 g/L is considered to be severe malnutrition and a cause of increased postoperative complications (2). 

These problems, and the fact that patients with esophageal cancer are unable to swallow during the early postoperative period, have always been challenging for surgeons. Gastrocolic malfunction after surgery, especially in the digestive system, impairs oral or gastric feeding for 2–5 days.

Iran has a high frequency of esophageal cancer. Therefore, we decided to compare the effects of early enteral feeding with parenteral feeding in the postoperative period among patients with esophageal cancer in terms of biochemical nutritional factors (total protein, albumin, transferrin, prealbumin) as well as immunological status (C3 and C4 complement levels) and inflammatory response (C-reactive protein [CRP] level) as well as postoperative complications during the time of hospitalization. 

The aim of the present study is to compare the efficacy of enteral and parenteral feeding methods in the postoperative period among patients with esophageal cancer.

## Materials and Methods

In this randomized clinical trial conducted between September 2008 and October 2009 at the Ghaem Hospital (Mashad, Iran), 40 patients with esophageal cancer were studied. Inclusion criteria were patients with middle or lower esophageal cancer (squamous cell carcinoma or adenocarc- inoma); operated using a transhiatal or transthoracic approach with a feeding jejunostomy. Exclusion criteria were cervical esophageal cancer; severe malnutrition before surgery (albumin <30 g/L; history of diseases affecting immunological function, such as diabetes or renal failure or chronic liver disease; history of using immunosuppressive drugs or corticosteroids; thoracic duct injury during surgery; history of preoperative radio- or chemo-radiotherapy (1-6).

Patients were randomly allocated into two groups (enteral and parenteral groups) using a computer-generated code. The average requirements in terms of energy, protein, carbohydrate and fat were calculated according to each patient, and the enteral and parenteral regimes were then designed based on the amount of macronutrients in the respective brand: Energy: 25–30 kcal/kg; protein: ~1.5 g/kg; fat:~30% of the energy intake; carbohydrate: the remaining energy requirement.

For parenteral nutrition, Intralipid (10%, Dextrose (10%) and aminoven (5%)) was used.

Age, gender, clinical signs and symptoms, serum nutritional proteins (total protein, albumin, transferrin, prealbumin), C3 and C4 complement levels (immunologic parameter) and high sensitivity (hs)-CRP (inflammatory parameter) were assessed. One day before surgery, blood tests were taken for routine preoperative measures as well as serum nutritional proteins, C3, C4 and hs-CRP levels. The patients then underwent surgery. From the first day after surgery, feeding was begun via jejunostomy or partial parenteral nutrition (PPN), according to the patient’s allocated group. The calorie intake was equal in both groups and on similar days. To prevent re-feeding syndrome (4), feeding was started with 500 kcal on the first day and gradually increased to 1,500 kcal by Day 5–7 after the surgery. Measuring of nutritional serum proteins and C3, C4 and CRP levels was repeated on Days 3 and 7 after surgery. 

Daily monitoring of patients was performed and data were collected regarding general condition, postoperative complications such as atelectasia, pneumonia, wound infections, fistula formation, return of bowel motion, defecation, gavage intolerance and the amount of chest tube drainage.

To obtain the precise calorie intake, a standard gavage solution with 1 kcal/cc energy was used (this solution comprised Nutric EN nutritional powder produced by Pooyan Co. Ltd).

 The calorie intake in the parenteral feeding group was calculated using standard solutions under the supervision of a nutritionist. Seven days after surgery, and after radiologic evaluation, oral feeding was started in both groups. 

***Statistical analysis***

Statistical analyses were performed using the SPSS statistical software package. Data analysis and statistical evaluation of quantitative variables was performed using a T-test for normal distribution of data and a Mann Whitney U-test in the case of non-normal distribution. A Chi-square or Fisher’s exact test was used for evaluation of categorical variables. A p-value <0.05 was considered as statistically significant. In the case that an independent variant was found to have interactive variants, a general linear model (likelihood test) was used in the subsequent process. 

## Results

Table 1 shows the patient characteristics. There was no significant difference between the two groups regarding age (mean), gender or body mass index. There was no significant difference between the two groups regarding the tumor location, surgical technique (P=0.65) or pathological staging (P=0.76). Blood loss in the two groups during surgery was not significantly different (P=0.85). 

Table 2 shows the results of total protein, albumin, transferrin and prealbumin levels in the two groups at different times before and after surgery. The results showed that there was no significant difference between the two groups.

Table 3 shows the results of C3, C4 and CRP levels in the two groups at different time points after surgery. There was a significant difference between the two groups in C3, C4 and CRP levels before and after surgery. Concentrations of C3 and C4 in the enteral feeding groups were significantly higher than those in the parenteral group, while CRP level was significantly higher in the parenteral group.The restoration of bowel movements (defecation and gas passing after surgery) in the enteral group occurred significantly sooner (4.5 days) than in the parenteral group (6 days), and the cost of treatment was significantly lower in the enteral group (gavage cost for each patient was 30,0000 Rls; almost $30) compared with the parenteral group (PPN cost was about 80,0000 Rls; almost $80) in one week. There was no significant difference between groups regarding complications or length of hospitalization (Table. 4). 

**Table 1:** Comparison of the baseline characteristics between groups

	**Enteral feeding group**	**Parenteral feeding group**	**P-value**
Gender (male/female)	9/11	12/8	0.21
Age (y)	64	57	0.25
Body mass index (k/m^2^)	17.6±9.7	18.1±4.9	0.75
**Tumor location & technique of surgery**			
Middle (Transthoracic esophagectomy)	12	11	0.65
Lower (Transhiatal esophagectomy)	8	9	
**Pathologic staging**			
- I	1	1	0.76
- IIA	8	9	
- IIB	2	2	
- III	9	8	

**Table 2:** Comparison the nutritional proteins levels between the two groups

	**Day 0**	**P-value**	**Day 3 after surgery**	**P-value**	**Day 7 after surgery**	**P-value**
Total protein						
Enteral F†	6.43±0.83	0.36	4.86±0.81	0.18	4.79±0.69	0.17
Parenteral Fǂ	6.67±0.86		5.73±1.04		5.36±0.80	
**Albumin**						
Enteral F	4.25±0.60	0.67	3.15±0.56	0.079	2.66±0.46	0.10
Parenteral F	4.33±0.52		3.27±0.65		3.08±0.52	
**Transferrin**						
Enteral F	193±74	0.167	104±28	0.057	95±51	0.720
Parenteral F	224±65		130±36		120±33	
**Prealbumin**						
Enteral F	15.0±8	0.915	10.6±85	0.065	14.5±12	0.216
Parenteral F	15.3±9		13.2±6.7		19±10	

† Enternal feeding

**Table 3:** Complement C3, Complement C_4_, CRP levels in study groups

	**Preoperative day**	**P-value**	**Day 3 after surgery**	**P-value**	**Day 7 after surgery**	**P-value**
Complement C3						
Enteral F	122±40	0.756	88±33	0.091	107±10	0.039
Parenteral F	127±54		82±35		92±12	
Complement **C**_4_						
Enteral F	27±10.5	0.748	24±6.8	0.037	25±7	0.046
Parenteral F	28±9		20±8.7		21±8	
**CRP**						
Enteral F	10±6	0.648	22±1.9	0.029	17±6.5	0.031
Parenteral F	9±5		27±1.1		20±7.3	

**Table 4:** Comparison of complications of the patients underwent esophagectomy in the two groups

	**Enteral feeding**	**Parenteral feeding**	**P-value**
Leakage from anastomosis	0	0	-
Pneumonia	1	1	-
Wound infection	0	0	-

In the group of patients who were fed by jejunostomy, gavage was well tolerated. In our study there was only one patient with abdominal distension and one case of diarrhea, occurring on Days 4 and 5 after surgery and due to excessive gavage volume. These complications were resolved by decreasing the volume and increasing the times of gavage. There was one death in the parenteral group, at Day 10 after surgery due to myocardial infarction. One patient was excluded from the study due to thoracic duct injury.

## Discussion

A previous study by Lassen et al. showed that earlier feeding from the first days after surgery on the upper digestive system did not increase the morbidity rate (5). Our results support these findings and suggest the potential of using gavage solutions immediately after major surgeries, including esophagectomy. In our study only one case of abdominal distension and one case of diarrhea (on Days 4 and 5 due to excessive gavage volume) were observed. 

Several studies have previously been conducted into the role of early enteral feeding versus parenteral feeding. 

Carr et al. confirmed that early feeding could increase intestinal mucosal permeability protein metabolism and the earlier restoration of bowel movement, and prevent postoperative infections (6). 

Gianotti et al. showed that early enteral feeding played no role in preventing complications and even caused complications such as abdominal distension, diarrhea and intolerance (7). Heyland et al. showed that early enteral feeding could cause earlier withdrawal of ventilators from very ill patients (8). 

Several studies have also been conducted assessing the volume of gavage solutions. In two large studies, Heyland et al. and Beier-Holgerson et al. recommended that the volume of gavage feeding at the first day after surgery should fall within the range of 480–1200cc and then be gradually increased (8,9). 

Our study began with a feeding volume of 500cc on the first day and was gradually increased up to 1,500cc on the fifth day to prevent the re-feeding syndrome.

For evaluation of the sufficiency and comparison of enteral and parenteral feeding Aiko et al. used several parameters, such as nutritional factors (total protein, albumin, transferrin, prealbumin) and effective immunological and inflammatory factors (C_3_, C_4 _and CRP levels) (2).

Serum levels of total protein, albumin, transferrin and prealbumin were used to evaluate nutritional status. Due to its shorter half-life, prealbumin is more responsive to nutrient absorption, and this is thought to be more useful in evaluating nutritional status (1,10).

In Japan, Aiko et al. (2000) (2) found no significant difference between groups fed using enteral or parenteral methods in terms of nutritional, immunologic or inflammatory indexes. In contrast, in our study there was no meaningful difference between the two groups regarding the serum protein levels, but C3 and C4 levels were significantly higher in the enterally-fed group. There was also a significant difference in hs-CRP levels between the two groups. 

Aiko et al., Baigrie et al. and Sand et al. showed that there were no significant differences between enteral and parenteral feeding in important complications such as pneumonia, fistula formation or wound infection.In all of these studies, the cost of parenteral feeding had been reported to be more than enteral feeding (11,12). In our study, the incidence of complications after surgery (pneumonia, fistula and wound infection) showed no meaningful difference between the two groups studied. In addition, we found that the cost of enteral feeding was significantly lower than that of parenteral feeding. 

## Conclusion

The results of our study show that enteral feeding can be effectively used in the first days after surgery, with few early complications and similar nutritional outcomes compared with the parenteral method. Furthermore, early enteral feeding can reduce the inflammatory response to the stress of surgery, improve immune response, lead to an earlier return of bowel movements, and involve a lower cost.
